# The role of mobile genetic elements in organic micropollutant degradation during biological wastewater treatment

**DOI:** 10.1016/j.wroa.2020.100065

**Published:** 2020-08-29

**Authors:** Ana B. Rios Miguel, Mike S.M. Jetten, Cornelia U. Welte

**Affiliations:** a Department of Microbiology, Institute for Water and Wetland Research, Radboud University, Heyendaalseweg 135, 6525, AJ Nijmegen, the Netherlands; b Soehngen Institute of Anaerobic Microbiology, Radboud University, Heyendaalseweg 135, 6525, AJ Nijmegen, the Netherlands

**Keywords:** Mobile genetic elements, Plasmids, Organic micropollutants, Wastewater treatment plants, Biodegradation, Bioaugmentation

## Abstract

Wastewater treatment plants (WWTPs) are crucial for producing clean effluents from polluting sources such as hospitals, industries, and municipalities. In recent decades, many new organic compounds have ended up in surface waters in concentrations that, while very low, cause (chronic) toxicity to countless organisms. These organic micropollutants (OMPs) are usually quite recalcitrant and not sufficiently removed during wastewater treatment. Microbial degradation plays a pivotal role in OMP conversion. Microorganisms can adapt their metabolism to the use of novel molecules via mutations and rearrangements of existing genes in new clusters. Many catabolic genes have been found adjacent to mobile genetic elements (MGEs), which provide a stable scaffold to host new catabolic pathways and spread these genes in the microbial community. These mobile systems could be engineered to enhance OMP degradation in WWTPs, and this review aims to summarize and better understand the role that MGEs might play in the degradation and wastewater treatment process. Available data about the presence of catabolic MGEs in WWTPs are reviewed, and current methods used to identify and measure MGEs in environmental samples are critically evaluated. Finally, examples of how these MGEs could be used to improve micropollutant degradation in WWTPs are outlined. In the near future, advances in the use of MGEs will hopefully enable us to apply selective augmentation strategies to improve OMP conversion in WWTPs.

## Highlights

• Organic Micropollutants (OMPs) are not fully removed by wastewater treatment plants (WWTPs).
• OMP-degrading genes tend to be linked to mobile genetic elements (MGEs).
• Most catabolic MGEs in WWTPs were found with culture-based methods.
• Culture-independent methods for MGE analysis are critically reviewed.
• Limitations of MGE-mediated bioaugmentation strategies in WWTPs are discussed.

## Graphical abstract

**Figure:**
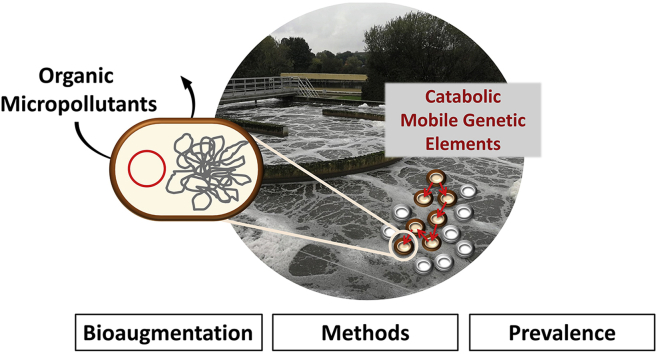

## 1 Introduction

### 1.1 Importance of wastewater treatment plants with emphasis on organic micropollutant removal

The World Health Organization estimates that the global water crisis claims approximately 3.5 million lives annually. These deaths are mainly caused by diarrheal diseases transmitted via feces-contaminated water and could easily be prevented by installing wastewater and drinking water treatment plants in affected areas (Kumar and Tortajada, 2020). Current biological wastewater treatment plants (WWTPs) in more developed countries are quite effective at removing the main pollutants from water: carbon, nitrogen, and phosphorous. However, their design is inadequate to remove the thousands of (toxic) organic compounds present at low concentrations that pose a risk to aquatic life and human health (Petrie et al., 2015; Rogowska et al., 2020). These compounds are called organic micropollutants (OMPs) because their concentrations in water range from nano- to micrograms per liter. OMPs are constantly released into the environment from a wide range of activities: industry (solvents), agriculture (pesticides, herbicides), the pharmaceutical industry and healthcare (drugs such as antibiotics or hormones), and households (personal care products). The diversity of chemical structures and release sources make OMP removal a great challenge for current water technology, leading to widespread investment in projects assessing and improving OMP removal by WWTPs as well as projects developing new quantification and detection methods (Dopp et al., 2019; Rizzo et al., 2019). New technological innovations in WWTPs are expected to contribute to reduce the impact of these pollutants and provide surface and groundwaters with minimal or preferably no OMPs.

### 1.2 Microbial degradation of organic micropollutants in wastewater treatment plants

**Fig. 1:**
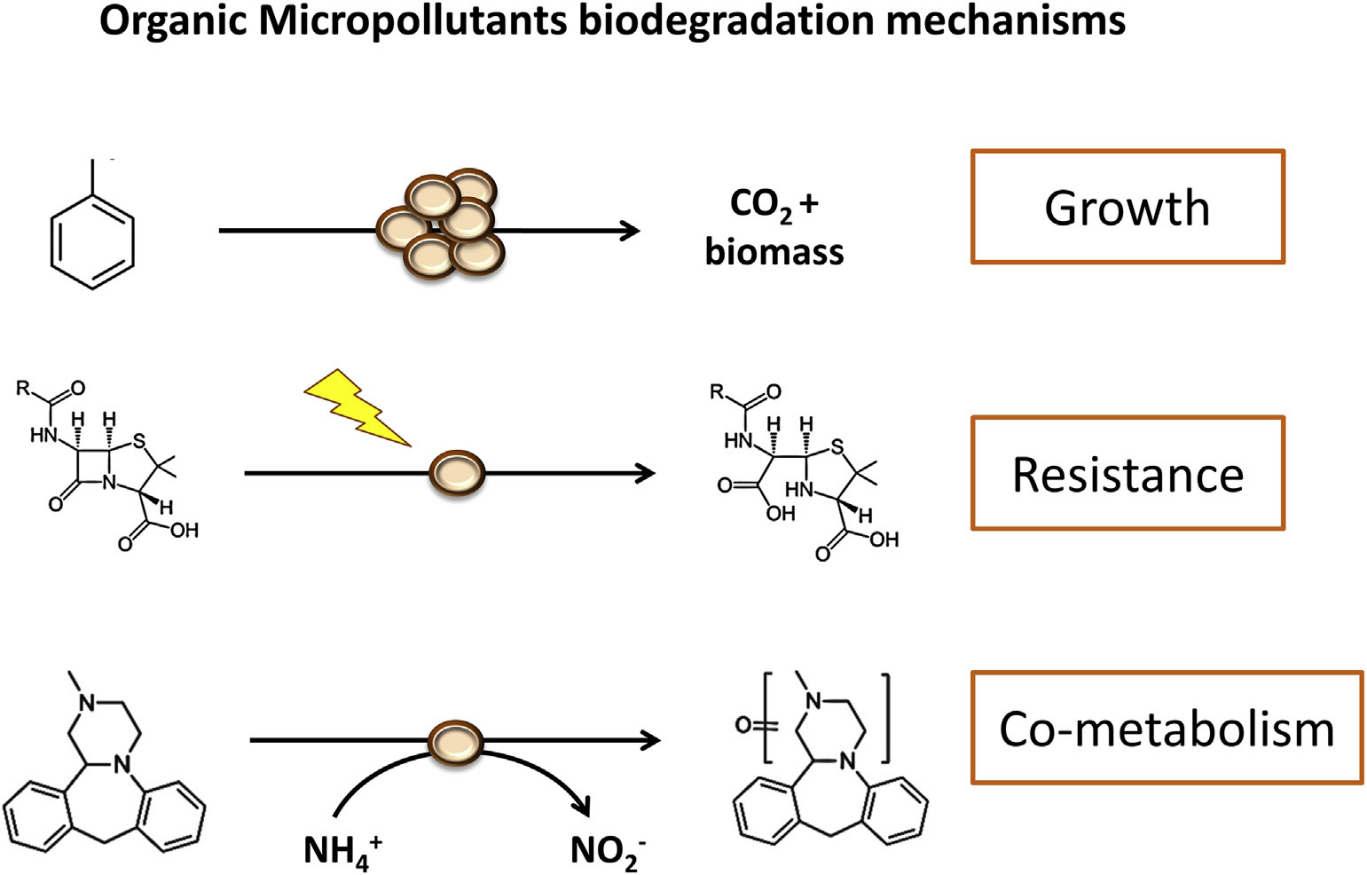
Organic micropollutants (OMPs) biodegradation mechanisms. Microorganisms can use OMPs as carbon and energy sources for growth (i.e. toluene), they can break the molecule to prevent toxicity (i.e. penicillin), and they can transform the molecule via co-metabolism, when a different growth substrate is being consumed (i.e. mianserin).

In addition to the physical and chemical techniques currently used in WWTPs to remove solids and pollutants, microorganisms continue to have a central role in wastewater treatment (Carey and Migliaccio, 2009). Microorganisms remove excess organic carbon, nitrogen, and phosphorous from waste streams (Wentzel and Ekama, 1997), but they have not been fully adapted and exploited to remove many OMPs. Only a few OMPs are completely degraded, and many others are partially removed (Gonzalez-Gil et al., 2018; Kleinsteuber et al., 2019). Among all biological removal pathways in WWTPs (biosorption, bioaccumulation etc.), the most promising for OMPs is biodegradation, which takes place through the following mechanisms (Fig. 1):

(i) Microorganisms can use OMPs as carbon and energy sources (e.g. toluene (Woods et al., 2011) and pesticides (Singh and Walker, 2006)). Complete OMP removal from water by conversion to CO_2_ gas or assimilation into solid biomass is the best scenario. Some micropollutants such as heavy metals do not contain carbon to assimilate, but microorganisms can still use these molecules as electron acceptors and remove them via precipitation (Tebo and Obraztsova, 1998).
(ii) Microorganisms can reduce the toxicity of OMPs by modifying their chemical structures by, for example, hydrolysis (e.g. antibiotics (Richmond and Sykes, 1973)). It is important to mention that not all antibiotic resistance genes (ARGs) encode for proteins that are able to degrade antibiotics. For example, many efflux pumps are able to provide resistance against toxic compounds without modifying their structures (Yelin and Kishony, 2018).
(iii) Microorganisms often transform OMPs in a process called co-metabolism (e.g. bisphenol, pesticides, pharmaceuticals, etc. (Fischer and Majewsky, 2014)). Co-metabolism occurs when OMPs undergo small structural transformations (e.g. hydroxylation, methylation, acetylation, etc.) during the consumption of other growth substrates by microorganisms. Such transformations occur when promiscuous or unspecific enzymes are able to utilize more than one substrate. These enzymes are not regulated to be active in the presence of the OMP, so in the absence of the growth substrate, the OMP cannot be transformed. A previous study observed this in a pure culture of the ammonia-oxidizing archaeon *Nitrososphaera gargensis*: the pharmaceutical miaserin was only transformed when ammonium was being oxidized (Men et al., 2016). The exact mechanism or enzymes involved in co-metabolism are oftentimes unknown although oxygenases are the main candidates under oxic conditions. The transformation products formed during co-metabolism can be accumulated in the extracellular medium (sometimes they are toxic for the cell) or be further transformed and utilized by the same or other microorganisms in a synergistic approach (Arp et al., 2001).

Various enzymes encoded in microbial genomes are good molecular effectors of OMP removal, such as β-lactamase for antibiotic breakdown and acetate kinase for co-metabolism (Richmond and Sykes, 1973; Gonzalez-Gil et al., 2017). Genes/proteins that are involved in central metabolism are typically encoded on the chromosome, while other complementary genes are located on mobile genetic elements (MGEs), which are DNA sequences encoding for proteins that mediate the movement of DNA within or between genomes (Frost et al., 2005). Genes encoded by MGEs may be useful to the organism only under transitory and occasional conditions, like the presence of toxic compounds or novel carbon sources (Tazzyman and Bonhoeffer, 2014).

### 1.3 Importance of mobile genetic elements in bacterial adaptation to organic micropollutants

**Fig. 2:**
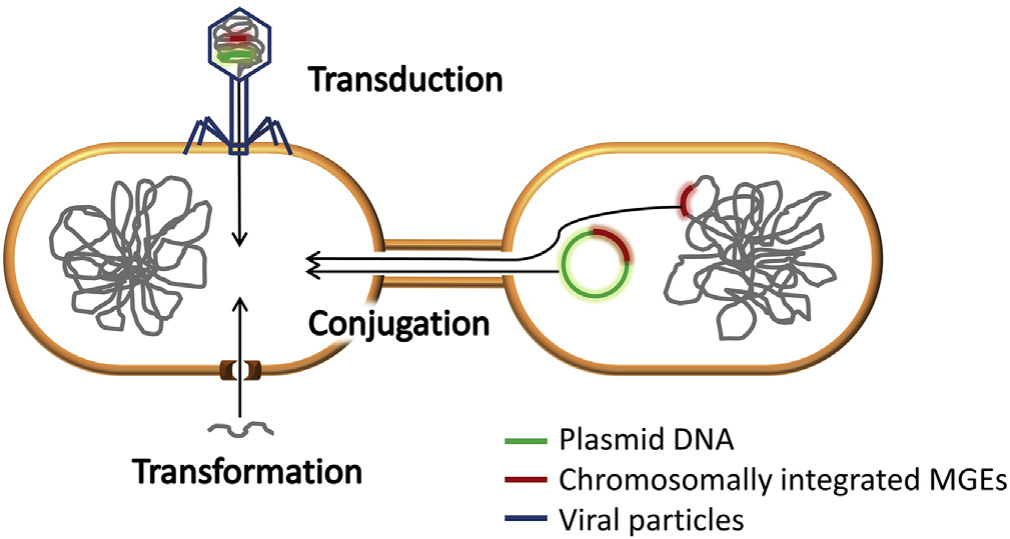
Representation of the three main horizontal gene transfer (HGT) mechanisms: conjugation via plasmids or chromosomally integrated mobile genetic elements (MGEs); transduction via viral particles; and transformation by competent cells able to take up naked DNA from the extracellular medium.

From an evolutionary perspective, OMPs have been recently introduced to the environment, so their degrading pathways are still under evolution. To make this happen, genes from different bacteria are combined in modules with the help of MGEs (van der Meer et al., 1992). Consequently, OMP-degrading genes are usually linked to extrachromosomal and chromosomal MGEs. The stable integration of these genes in the chromosome will occur if OMPs become the main energy and carbon sources for some bacteria in environments with a constant and high OMP concentration (Tazzyman and Bonhoeffer, 2014). This is not the current situation in WWTPs, so MGEs still play a crucial role in OMP biodegradation. MGEs are involved in gene movement between non-related microorganisms, which is called horizontal gene transfer (HGT) and is mediated by three main mechanisms: conjugation, transduction, and transformation (Fig. 2) (Dröge et al., 1999). Conjugation is performed by cell-to-cell contact and requires MGEs such as chromosomally integrated transposons or conjugative plasmids. Transduction is the process by which ‘foreign’ DNA is introduced into a microbial cell with the help of a viral vector such as a bacteriophage. During transformation, naturally or artificially prepared competent cells take up free DNA from the environment (Frost et al., 2005). The best-studied form of HGT is conjugation, and the spread of MGEs harboring ARGs in WWTPs and other environments has received extensive attention (Heuer et al., 2012; Guo et al., 2017; Jiao et al., 2017).

Given these characteristics, MGEs may play a relevant role in WWTPs, where microorganisms are in contact with OMPs and in close proximity to each other. Several ‘catabolic’ MGEs have been discovered in WWTPs, and some preliminary studies have succeeded in improving the removal of contaminants by increasing HGT of genes involved in their degradation. Consequently, MGEs represent a promising tool for engineering the bacterial communities in WWTPs towards the biodegradation of OMPs. Before such a scenario can be implemented, additional insights on the prevalence and role of catabolic MGEs in WWTPs are needed. This review summarizes the different OMP-transforming genes located on MGEs in WWTPs and the tools for analyzing MGEs in complex environmental samples. Furthermore, attempts to engineer and stimulate conjugation under WWTP conditions are critically reviewed in order to reveal the main limitations that need to be overcome in future treatment methods.

## 2 Catabolic mobile genetic elements in wastewater treatment plants

Many genes responsible for the catabolism of OMPs in WWTPs are located on MGEs such as plasmids, transposons, and insertion sequences (Top and Springael, 2003; Nojiri et al., 2004; Dunon et al., 2018). Transposons and insertion sequences can be integrated into the chromosome or “jump” into plasmids, which are extrachromosomal DNA fragments that are able to autoreplicate (Osborn and Böltner, 2002). Some of these MGEs can be transferred to other cells during conjugation. For this process, MGEs use their own conjugative genes (self-transmissible) or hijack the conjugative systems of other elements (Osborn and Böltner, 2002; Smillie et al., 2010; Carraro et al., 2017). MGE mobility produces mutations and rearrangements of genetic material inside microbial cells. As a result, the associated genes can form new functional clusters and spread rapidly through the bacterial population, subsequently providing their hosts with advantages such as resistance to toxic compounds or the ability to exploit alternative carbon sources.

MGEs accelerate microbial adaptation to the transformation of new chemicals (Top and Springael, 2003), so they might be actively involved in OMP removal in WWTPs. Several studies have found similar catabolic gene clusters in phylogenetically unrelated strains isolated from geographically distant soils and WWTPs (van der Meer et al., 1992; Williams and Sayers, 1994; Ouchiyama et al., 1998; Shintani et al., 2005). For example, the upper part of the carbazole degradation pathway from *Pseudomonas* sp. isolated from a WWTP in Tokyo contains similar genes to other strains isolated from a different WWTP more than a 1000 km away, and from farm soil. On the other hand, the lower part of this pathway degrading catechol is highly similar to the one present in plasmids found all around the world: TOL plasmid pWW0, plasmid NAH7, and plasmid pVI150. This shows the way degradation pathways are usually formed: by combining genes coming from different microorganisms or genetic backgrounds. This can also be proven by the different C + G content of plasmids and chromosomes in many microorganisms. Furthermore, a few experiments have measured correlations between MGEs and the presence of OMPs. In the first part of this section, we will list catabolic MGEs found in WWTPs and discuss biases underlying these discoveries. In addition, studies linking MGE abundance to pesticide turnover in agricultural WWTPs will be examined, and gaps in the knowledge of the contribution of catabolic MGEs to wastewater treatment will be highlighted.

### 2.1 Prevalence of catabolic mobile genetic elements in wastewater treatment plants

#### 2.1.1 Conjugation

The most studied HGT route in WWTPs is plasmid transfer via conjugation. Plasmids consist of extrachromosomal DNA and are able to autoreplicate and sometimes automobilize. Enzymes involved in the degradation of different pollutants are often encoded on plasmids, and thus all of the required genes of the degradation pathway can be transferred together in one HGT event. Consequently, catabolic plasmids are relatively large and mostly low copy number, which decreases the energy required to replicate the plasmid (Top and Springael, 2003). Genes encoding enzymes for degrading man-made compounds are usually located on more promiscuous plasmids, meaning their host range is phylogenetically wider, (incompatibility group IncP-1 plasmids) than genes encoding enzymes that degrade naturally occurring organic compounds (incompatibility group plasmids IncP-2, IncP-9) (Nojiri et al., 2004). Therefore, IncP-1 plasmids might efficiently harbor and distribute genes for the degradation of new pollutants. Plasmids in the same incompatibility groups are not able to coexist together in a cell because they usually compete for the same replication factors. Although WWTPs often contain many OMPs, a plasmid metagenomic study suggested that IncP-1 plasmids occur less frequently than other plasmid types such as IncF in WWTPs (Schlüter et al., 2008). Moreover, the majority of plasmids seemed to be non-self-transmissible, so IncP-1 plasmids might act as helping vectors by assisting the transfer of other non-conjugative plasmids.

MGEs such as transposons or insertion sequences can also be found on plasmids. These MGEs are able to shuffle genes in the chromosome and thus help create novel catabolic pathways. When they are further combined with promiscuous plasmids, they represent a very efficient vector to spread new catabolic capabilities (Ma et al., 2007; Bahl et al., 2009). IS*1071* and IS*6100* are the most frequent insertion sequences associated with OMP degradation (Kato et al., 1994; Sota et al., 2006; Gai et al., 2010; Takeo et al., 2012; Dunon et al., 2013; Martini et al., 2015). These insertion sequences are found in many catabolic plasmids such as pTSA and pCNB1 flanking degradation genes and form transposon modules in which the whole DNA sequence included between the insertion sequences is transferred as a unit. Thus, insertion sequences provide a solid structure for efficient recruitment of catabolic genes to plasmids and dissemination of these genes among bacteria.

Table 1 lists several catabolic plasmids found in WWTPs across the world. These plasmids mostly belong to the IncP-1β incompatibility group, are conjugative and are approximately 60–100 kb. Although IncP-1 plasmids appear to be the most abundant plasmids containing OMP-degrading genes in WWTPs, they are overall less abundant than other plasmid types based on a previous metagenome analysis (Schlüter et al., 2008). Most bacteria containing IncP-1 plasmids are affiliated with the order *Burkholderiales* of the class Betaproteobacteria, a metabolically highly diverse group that plays a significant role in nitrogen and carbon removal in WWTPs due to their capability of heterotrophic denitrification (Zielińska et al., 2016; Fan et al., 2017). *Burkholderiales* are also known to degrade a wide range of aromatic compounds because of the large variety of oxygenase enzymes they encode (Perez-Pantoja et al., 2012; Pereira et al., 2014). Furthermore, some members of this order are opportunistic pathogens that are resistant to antibiotics (Bilgin et al., 2015). This creates controversy when using OMP-degrading microorganisms in bioaugmentation strategies. Each catabolic plasmid confers novel degradation properties to its host: many are able to use different halogenated and aromatic compounds as carbon and energy sources. Several transposons and insertion sequences have been linked to these catabolic genes, but by far the most abundant in culture-based studies is IS*1071*.

**Table 1:** Catabolic MGEs isolated from several WWTPs around the world.

**Plasmid/MGE**	Accession number (GenBank)	Plasmid type/size (kb)	Catabolic genes	Substrates	Host bacteria	Isolation site	Analysis tools	Ref.
**pA81/ TnAx*I* (ISAx*I*a & ISAx*I*b)**	NC_014641.1	IncP1β Conjugative 98	*OhbRAB, mocpRABCD, hybRABCD*	Chlorobenzoates: 2-chlorobenzoate & 2,5-dichlorobenzoate. Salicylate	*Achromobacter xylosoxidans* A8	Sewage treatment plant in Hong Kong	Qiagen Kit + DNase + TRACA	(Jencova et al., 2008; Zhang et al., 2011)
**pUO1/ IS*1071,* Tn*Had1 &* Tn*Had2***	NC_005088.1	Conjugative 65	*dehH1* & *dehH2*	Halogenated compounds: fluoroacetate & chloro-, bromo-, & iodoacetate	*Delftia acidovorans* B (*Moraxella* sp. strain B)	Fluoroacetate-containing industrial WWTP in Japan	Culture	Sota et al. (2002)
**pGNB1, pKV11, pKV29, pKV36/ IS*1071***	EF628291.1; JN648092.1; JN648090.1; JN648091.1	IncP1β Conjugative 60–70	*ldpA & tmr*	Triphenylmethane dyes: crystal violet, malachite green, basic fuchsin	*Comamonas* sp. H4-3; *Delftia; Comamonas* sp. H4-3	WWTP in Germany	Culture with crystal violet	(Schlüter et al., 2007; Stolze et al., 2012)
**pTSA/ IS*1071*** **pPSB/ IS*1071***	AH010657.3	IncP1β Conjugative 85	*tsaMBCD* & *psbA(C)*	Arylsulfonates: toluenesulfonate and benzosulfonate	*Comamonas testosteroni* T-2 and PSB-4	Urban and industrial sewage treatment plants	Culture with aromatic sulfonates	(Thurnheer et al., 1986; Junker et al., 1996; Junker and Cook, 1997; Tralau et al., 2001)
**pNB8c/ IS*1071***	JF274990.2	IncP-1 Conjugative 100	*tdnQ*	Aniline, 3-chloroaniline not completely (to 4-chlorocatechol)	*Delftia acidovorans* B8c	WWTP of a potato-processing company in Belgium	Culture with aniline and 3-chloroaniline	(Boon et al., 2001)
**pI2**	JF274989.1	IncP-1β 100	*tdnQ*	Aniline, 3-chloroaniline not completely (to 4-chlorocatechol)	*Comamonas testosteroni* I2	Domestic WWTP in Belgium	Culture with aniline and 3-chloroaniline	(Boon et al., 2000; Wu et al., 2015)
**pCNB1/ IS*1071***	EF079106.1	IncP-1β Conjugative 91.2	*cnbBARCabDEFGH*	4-Chloronitrobenzene	*Comamonas* sp. Strain CNB-1	WWTP from a CNB production factory in China	Culture with 4-chloronitrobenzene	(Wu et al., 2005, Wu et al., 2006; Ma et al., 2007)
**pND6-1/*tnp*A1, *tnp*A2, *tnp*A3**	NZ_AY208917.1	IncP-7 102	*nahAaAbAcAdBFCED* + *nahGTHINLOMKJY*	Naphthalene	*Pseudomonas* sp. strain ND6	Industrial wastewater in China	Culture	Li et al. (2004)
**pAC25**		Conjugative 117		3-Chlorobenzoate	*Pseudomonas putida* AC858	Sewage plant in New York, US	Culture	(Chatterjee et al., 1981)
***clc* element** ***intB13***	AJ004950.1 (integrase gene)	Conjugative 105	*clcABDE*	3-Chlorobenzoate	*Pseudomonas* sp. *strain B13*	Sewage plant in Germany	Culture with 3-chlorobenzoate	(Dorn et al., 1974; van der Meer et al., 2001)
**pCAR1/IS*5car1*-IS*5car4*,** **IS*Pre1*, IS*Psp4*, IS*Ppu7*,** **Tn*4676***	NC_004444.1	IncP-7 Conjugative 199	*CarAaBaBbCAcORF7AdDFE*	Carbazole/dioxin	*Pseudomonas* spp*.CA10, K23, K22, K15, OM1*	Activated sludge in Japan	Culture with carbazole	(Ouchiyama et al., 1993, 1998; Nojiri et al., 2001; Inoue et al., 2004; Shintani et al., 2005)
**pAtC58** a	AF283811.2	542	*socRABCD*	Opine: deoxy-fructosyl-glutamine	*Agrobacterium tumefaciens*	WWTP in Germany	Culture with antibiotics	Schlüter et al. (2008)
**pL6.5** a	AJ250853.1	Non-conjugative 105.5	–	Toluate	*Pseudomonas fluorescens* L6.5	WWTP in Germany	Culture with antibiotics	Schlüter et al. (2008)
**pCAR1/ TnpAb** a	NC_004444.1	IncP-7 Conjugative 199	*car* & *ant*	Carbazole/dioxin	*Pseudomonas resinovorans* CA10	WWTP in Germany	Culture with antibiotics	Schlüter et al. (2008)

a Plasmids were not isolated. Only plasmid markers were sequenced from a WWTP plasmidome sample.

One drawback of the data in Table 1 is that most of it comes from culture-dependent methods. Thus, the results might not represent the actual situation in WWTPs due to the “great plate count anomaly”, in which only a fraction of the true microbial diversity can be captured (Harwani, 2013). Culture-independent methods provide a more comprehensive characterization of the catabolic MGEs’ diversity in WWTPs. However, culture-dependent methods cannot be totally substituted since they are necessary to study the function of the highly abundant unannotated genes linked to MGEs. The last three plasmids in Table 1 were found in a metagenomic study. However, WWTPs samples in that study were first cultivated with several antibiotics, and then all plasmids (the ‘plasmidome’) from the resistant community were sequenced. Plasmid replication proteins of catabolic plasmids pAtC58 and pL6.5 were identified. The transposon TnpAb, previously associated with pCAR1, was also observed (Schlüter et al., 2008). Another metagenomic analysis performed on activated sludge from two WWTPs in Hong Kong identified genes for several OMP-degrading enzymes (Fang et al., 2013). Up to 40% of these genes were encoded on plasmids. In that study, activated sludge samples were not precultured, thus providing a more realistic picture of catabolic MGEs in WWTPs biased only by DNA extraction and bioinformatic methods.

Several metagenome analyses of WWTPs have specifically looked for MGEs transferring ARGs due to the emergent risk they pose to human health (Li et al., 2015; Guo et al., 2017; Che et al., 2019; Pazda et al., 2019). However, the link between MGEs and genes encoding OMP-degrading enzymes in metagenomes has not attracted the same attention and only few studies investigated this (see previous paragraph (Fang et al., 2013)). This might be due to the incompleteness of the respective gene databases. EAWAG-BBD (http://eawag-bbd.ethz.ch/) is the most up to date database to study microbial degradation of OMPs (Ellis and Wackett, 2012), but many genes encoding OMP-degrading enzymes are still not known or may not even have evolved yet. Furthermore, identifying MGEs and the genes associated with them in environmental samples is still a challenge, as will be further discussed in the methods section.

#### 2.1.2 Transduction

Transduction is often considered the most efficient way of HGT between different biomes: it does not require physical contact between bacteria, in contrast to conjugation, and phage particles protect the DNA from degradation, in contrast to transformation. Furthermore, transduction occurs in a wide range of bacteria at higher frequencies than previously thought. Any kind of bacterial DNA can be packed inside a bacteriophage capsid, including chromosomal fragments and MGEs such as plasmids, transposons or insertion sequences (Fig. 2) (Muniesa et al., 2011). This can happen by two different mechanisms: generalized and specialized transduction. During the first one, the virus hydrolyses the host cell DNA and any fragment is able to be incorporated into the virus capsid. When the virus infects a different cell, the non-viral DNA undergoes homologous recombination forming a recombinant cell. In specialized transduction, the viral genome gets integrated into the host cell genome. During excision, it sometimes takes the cell’s genes flanking the virus integrated site. Thus, only those genes adjacent to the integration site are able to be transferred to a different cell. The size of DNA fragments that can be packed inside a bacteriophage particle is limited by the capsid size, with an average size of 50 kb and maximum size of 100 kb (Muniesa et al., 2013).

The study of viral communities in WWTPs has been limited due to the low percentage of host bacteria that can be cultured in the laboratory. However, recent advances in high-throughput sequencing technologies have enabled researchers to sequence the whole viral metagenome in several samples (Edwards and Rohwer, 2005; Pérez-Losada et al., 2020). Genes identified in the phage metagenomes of several WWTPs and other environments such as the mouse gut include ARGs and 16S rRNA genes from Firmicutes, Proteobacteria, Bacteroidetes, and Actinobacteria (Parsley et al., 2010a, Parsley et al., 2010b; Del Casale et al., 2011; Modi et al., 2013). Interestingly, *Nitrospira* and Planctomycetes genes were not found in the clones of a WWTP phage metagenome, suggesting that these phyla are not highly involved in transduction (Del Casale et al., 2011).

OMP-degrading genes have not been extensively explored in bacteriophage communities. In soil with prior pesticide exposure, a chlorohydrolase gene responsible for the dehalogenation of atrazine was found in the viral metagenome (Ghosh et al., 2008). In addition, the phage metagenome from an activated sludge sample was sequenced and subsequently analyzed to determine the functions of the different open reading frames (ORFs) or protein-coding genes (Parsley et al., 2010b). The most abundant subsystem was related to the metabolism of macromolecules such as carbohydrates, proteins (and amino acids), and lipids. Furthermore, 5% of the ORFs were related to aromatic compound metabolism. Approaches similar to those used to identify ARGs inside viral particles in WWTPs (Parsley et al., 2010a, Parsley et al., 2010b; Muniesa et al., 2011) could be adopted to find specific OMP-degrading functions in future work.

#### 2.1.3 Transformation

Naturally competent bacterial cells can gain new functions by taking up extracellular DNA (exDNA). This kind of DNA is released to the environment via two main mechanisms: bacterial cell lysis and active secretion. Cell lysis can be the result of a bacteriophage infection, autolysins or reactive oxygen species. Active secretion of DNA is usually performed by the type IV secretion apparatus although vesicle-mediated exDNA delivery has also been demonstrated in some microorganisms such as *Streptococcus pneumoniae* (Sarkar, 2020; Wang et al., 2020a). Apart from its role in HGT, exDNA has a structural function in microbial aggregates such as biofilms and small-sized aerobic granular sludge. This has been proven by adding exogenous DNases and observing bacterial detachment and disruption of biofilms and small aerobic granules (Jakubovics et al., 2013; Wang et al., 2020b). Furthermore, adsorbed DNA is transformed at higher rates than free DNA, making bacterial aggregates a good spot for transformation due to the high amounts of extracellular material where DNA can be attached (Dong et al., 2019). Several studies of exDNA in WWTPs have shown the high abundance of extracellular MGEs (65% of the exDNA) (Calderón-Franco et al., 2020) and the presence of several ARGs (Dong et al., 2019; Yuan et al., 2019; Zhou et al., 2019). However, nothing is currently known about the OMP-degrading genes present in exDNA in WWTPs. Natural transformation is known to be a driving force of evolution and it is logical to think that it plays a role in the creation of novel degradative pathways. For that reason, new studies looking for OMP-degrading genes in exDNA from WWTPs are needed.

### 2.2 Correlation between mobile genetic elements and organic micropollutants abundance

Only a few studies have attempted to elucidate the direct contribution of catabolic MGEs to OMP removal during wastewater treatment. These studies have been performed in on-farm biopurification systems (BPSs), installations that treat pesticide-contaminated wastewater on farms. One study compared pesticide-treated and non-treated soils and BPSs by measuring the abundance of IncP-1 plasmids and IS*1071* insertion sequences (Dunon et al., 2013). The authors subsequently sequenced the accessory genes between two IS*1071* sequences (Dunon et al., 2018). Interestingly, they observed a correlation between pesticide exposure and IncP-1/IS*1071* abundance and were able to identify several biodegradation genes flanked by IS*1071* sequences. This demonstrates the importance of IncP-1 plasmids and IS*1071* in bacterial community adaptation to pesticides.

A different study monitored the abundance of different types of MGEs and the concentrations of different pesticides in a BPS for an entire year (Dealtry et al., 2014). They observed a correlation between the presence of the analyzed OMPs and the abundance of IncP-1β plasmids and intI2 elements, another kind of MGE able to capture, express and spread genes. These results also point towards a role of specific MGEs in microbial adaptation and subsequent pesticide degradation in BPSs. These catabolic MGEs might also have the potential to facilitate OMP degradation in urban WWTPs, but new studies of catabolic MGE function and dynamics in WWTPs are needed.

## 3 Methods for mobile genetic elements analysis in environmental samples

**Fig. 3:**
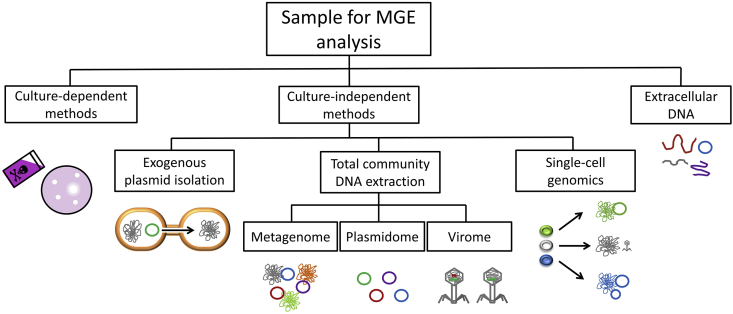
Methods for mobile genetic elements (MGEs) analysis in environmental samples. First, methods can be classified according to whether or not samples are precultured in the lab before MGE identification. When employing culture-independent methods, MGEs (mostly plasmids) can be first transferred to a recipient cell (exogenous plasmid isolation) or identified after total microbial community DNA extraction or using single-cell technologies. Several protocols for purifying plasmid DNA or viral particles before high-throughput sequencing are available. Finally, DNA present in the extracellular medium can be sequenced. Extracellular DNA has the potencial to perform HGT to competent cells, so all of it can be considered a MGE. For a thorough comparison of these methods see Table 2 and Box 1.

Studies providing an overview of catabolic MGEs in WWTPs are scarce, most likely due to the lack of knowledge in current degradative gene databases and technical limitations. WWTP samples consist of complex microbial communities and a large variety of organic matter, which can interfere with protocols for analyzing MGEs. Various strategies can be applied to obtain information about MGEs together with their associated genes in WWTPs (Fig. 3, Table 2), and the choice mainly depends on the study goals. Each method has different biases in terms of plasmid sizes, traits or hosts, and knowing the advantages and limitations of each method is of great importance for achieving the specific research goal (Table 2, Box 1).

**Table 2:** Strengths and limitations of methods analyzing MGEs in complex environmental samples.

MGE analysis methods			Strengths	Limitations	Ref.
Culture-dependent			• The function of MGEs and accessory genes can be studied. • MGEs and host cells can be linked.	• It does not represent the real MGE diversity in an specific environment because only a few microorganisms can be cultured.	Schlüter et al. (2007)
Culture-independent	Exogenous plasmid isolation		• It covers more plasmid diversity than culture-dependent methods.	• No host cells can be identified. • Only mobilizable plasmids compatible with the recipient strain can be identified. • It does not include chromosomally integrated MGEs.	(Dröge et al., 1999; Smalla et al., 2015)
	Total community DNA extraction	Metagenomics (short read sequencing)	• It covers more MGE diversity than culture-dependent and exogenous plasmid isolation. • High sequence accuracy	• No host cells can be identified. • Difficult to assemble and identify MGEs due to repetitive sequences. • PCR amplification coverage bias	Arredondo-Alonso et al. (2017)
		Plasmid metagenomics	• It facilitates the study (assembly) of plasmids and virus compared to metagenomics by decreasing the complexity of the sample.	• No host cells can be identified. • It does not include chromosomally integrated MGEs. • Plasmid and virus isolation and amplification (PCR, MDA, TRACA) methods are biased towards small plasmids.	(Jones and Marchesi, 2007; Thurber et al., 2009; Sentchilo et al., 2013; Smalla et al., 2015)
		Viral metagenomics			(Edwards and Rohwer, 2005; Thurber et al., 2009; Parsley et al., 2010a, Parsley et al., 2010b; Pérez-Losada et al., 2020)
	Single-cell analysis		• MGEs and host cells can be linked. • The assembly of MGEs becomes easier by decreasing the complexity of the sample. • High resolution in distinguishing strains from the same species	• PCR amplification coverage biases (overcome by direct library preparation) • High price	(Zhang and Liu, 2008; Rinke et al., 2014; Spencer et al., 2016; Doud and Woyke, 2017; Lan et al., 2017)
	(Improvements to short read sequencing)	Long-Read sequencing (Oxford Nanopore and PacBio single-molecule real-time (SMRT))	• MGEs and host cells can be linked (only in SMRT using nucleotide methylation information). • The assembly of MGEs becomes easier adding long-read information.	• High error rate (used in combination to short-read sequencing) • Limited throughput • High price	(Rhoads and Au, 2015; Jain et al., 2016; Beaulaurier et al., 2018)
		Hi-C	• MGEs and host cells can be linked. • The assembly of MGEs becomes easier adding physical DNA interactions information.	• PCR amplification produce biases by underrepresenting genomic short-distance ligations (overcame by SAFE Hi-C) • High price	(Van Berkum et al., 2010; Burton et al., 2014, Niu et al., 2019)
		Optical mapping	• The assembly of MGEs becomes easier by adding long-range information.	• Low resolution (1 kb approximately compared to the single base pair resolution of sequencing) • No host cells can be identified.	Michaeli & Ebenstein (2012)
		Link reads		• Repetitive sequences limit accuracy and coverage (not so much research done to identify biases of Link reads) • No host cells can be identified.	Ott et al. (2018)
Extracellular DNA isolation	Magnetic bead adsorption		• Low volume sample (5 mL) • Better DNA quality and yields compared to other isolation methods.	• Cell lysis not checked	Yuan et al. (2019)
	Anion-exchange chromatography		• No cell lysis • Higher DNA yields compared to other isolation methods.	• Higher sample volume to obtain high quality DNA (1000 mL)	Calderón-Franco et al. (2020)

Box 1 **Strengths and limitations of methods analyzing mobile genetic elements (MGEs) in complex environmental samples.**

The method of choice to analyze MGEs in environmental samples depends on the study objective. Each technique has its own biases and it is important to take them into account before designing a new experiment. Table 2 explains the strengths and limitations of the methods described in this review. Overall, culture-independent methods provide a more comprehensive and diverse picture of MGEs in a specific environment, but culture-dependent methods are still necessary to study the function of unknown MGEs and genes. Furthermore, culture-dependent methods are able to link MGEs and host cells, while only some culture-independent methods are able to do that: single-cell, Hi-C, and single-molecule real-time (SMRT) technologies. The majority of culture-independent methods rely on total community DNA extraction for further sequencing. The main challenge of short-read sequencing is the correct assembly of MGEs due their long repetitive sequences and the sample complexity (Arredondo-Alonso et al., 2017). In order to decrease the complexity of the sample, plasmids or phages can be isolated before sequencing (Jones and Marchesi, 2007; Thurber et al., 2009; Sentchilo et al., 2013). This creates additional biases depending on the isolation and amplification method, which usually enrich small plasmids. Furthermore, single-cell technologies are able to simplify the process by sequencing single cells from the complex sample (Zhang and Liu, 2008; Rinke et al., 2014; Doud and Woyke, 2017; Lan et al., 2017). A more accurate assembly of repetitive sequences can be done with technologies that add long-range information or physical DNA interactions information to the sequencing process. Long-read sequencing technologies such as Oxford Nanopore and PacBio SMRT sequence long DNA molecules with the disadvantage of having high error rates and limited throughput (Rhoads and Au, 2015; Jain et al., 2016). Optical mapping is a fast and cheap method able to provide long-range information such as the presence of specific sequences, nucleotides, or AT-GC regions in a DNA sequence (Michaeli and Ebenstein, 2012). However, it has low resolution meaning it only identifies a specific footprint, not a detailed sequence of the DNA molecule like long-read sequencing. Finally, the linked read technology barcodes reads belonging to a long sequence so that they will be assembled together after sequencing (Ott et al., 2018). These three techniques are usually combined with short-read sequencing to increase the sequence accuracy. On the other hand, Hi-C technology uses the physical DNA interactions between parts of the chromosome or between the chromosome and MGEs in order to improve the assembly of metagenomes and link MGEs to host cells (Van Berkum et al., 2010; Burton et al., 2014). Similar to other technologies such as short-read sequencing and single-cell sequencing, Hi-C harbors the bias of PCR amplification. However, this problem is currently overcome by SAFE Hi-C (Niu et al., 2019). Finally, if the objective is to analyze extracellular DNA (exDNA), two recent methods have shown increased DNA quality and yields in wastewater samples. The first one consists of exDNA adsorption to magnetic meads and has the advantage of a low sample volume (5 mL) (Yuan et al., 2019). The second method consists of exDNA adsorption and elution in an anion-exchange chromatography column. With this method higher sample volumes are needed (1000 mL) (Calderón-Franco et al., 2020).

Alt-text: Box 1

The majority of studies investigating catabolic plasmids in WWTPs have used culture-dependent methods targeting the degradation of a specific compound (Table 1). This approach consists of plating an environmental sample onto a selective medium to isolate cells and/or MGEs with desired characteristics, e.g. antibiotic resistance or crystal violet degradation (Schlüter et al., 2007, 2008). Plasmids or other MGEs present in the genomes of the cultured community members can subsequently be analyzed by different molecular techniques (PCR, hybridization, sequencing) and linked to specific bacterial strains or associated genes. However, if the aim is to obtain a representation of all catabolic MGEs present in the WWTP, this approach would not be ideal because only a small proportion of the WWTP microbial community can be cultured (Harwani, 2013).

To obtain a more representative overview of MGE abundance and OMP degradation in WWTPs, culture-independent methods might be more appropriate. Exogenous plasmid isolation permits the identification of transferable plasmids in a sample without cultivation of the original plasmid host (Dröge et al., 1999; Smalla et al., 2015). In this method, bacterial communities in the sample are mixed with recipient cells to allow DNA transfer. The mixture of cells is then resuspended on selective media to isolate the recipient cells that have obtained the target capacity. The cells can also be plated on rich media to identify all transferred plasmids. In this approach, only mobilizable plasmids compatible with the selected recipient are characterized. One shortcoming is that it is not possible to identify the original plasmid host.

A less biased approach consists of total DNA isolation from a sample and direct sequencing of the isolated DNA. However, MGEs such as plasmids and viruses are very difficult to reconstruct and identify in metagenomic analyses because they possess long repetitive zones, especially large plasmids (Arredondo-Alonso et al., 2017). Many bioinformatic tools have been recently developed to overcome this challenge in short-read sequencing. Examples of them are PlasmidFinder, cBar, PLACNET, (meta)plasmidSPAdes, recycler, plasflow, MOB-suite, mlplasmids, plaScope, gplas, plasClass, plasGUN,SCAPP, metaviralSPAdes (Zhou and Xu, 2010; Carattoli et al., 2014; Rozov et al., 2017; Vielva et al., 2017; Arredondo-Alonso et al., 2018; Arredondo-Alonso et al., 2020; Krawczyk et al., 2018; Robertson and Nash, 2018; Royer et al., 2018; Antipov et al., 2019, 2020; Fang et al., 2020; Pellow et al., 2020a, Pellow et al., 2020b; Pellow et al., 2020, 2020). These tools identify plasmids and viruses in metagenomic samples or bacterial isolates in a variety of ways, ranging from the simpler approach of blasting against a plasmid database (PlasmidFinder, MOB-suite) to the use of more complex deep neural networks (cBar, PlasFlow). Moreover, algorithms such as Recycler, metaplasmidSPAdes, metaviralSPAdes and SCAPP use the assembly graphs to identify regions of different coverage, which are the MGEs. Another option to further improve plasmid identification is long-read sequencing technologies such as Oxford Nanopore or PacBio Technologies (Rhoads and Au, 2015; Jain et al., 2016). However, short-read sequencing is still used in combination with long-read sequencing in order to correct the sequence errors of the latter technique (Amarasinghe et al., 2020). Sequencing has also been used in combination to other methods in order to overcome the assembly challenge of long repetitive DNA areas: linked reads, optical mapping, and proximity ligation technologies. The linked read strategy barcodes all small sequences coming from the same long-sequence and then accurate short-read sequencing is performed (Ott et al., 2018). In optical mapping, long DNA molecules are fluorescently labelled at specific sequences and stretched in order to determine the distances between labels with a fluorescence microscope. It has low resolution so it is better used in combination with short-read sequencing too (Michaeli and Ebenstein, 2012). Finally, proximity ligation technologies such as Hi-C use the information of physical DNA interactions inside the cell to help assemble MGEs (Van Berkum et al., 2010).

Instead of sequencing total DNA, many studies have tried to enrich plasmids or viral particles before sequencing. All plasmids in a microbial community sample can be separated from chromosomal DNA by alkaline lysis, followed by size-dependent DNA separation methods such as ultracentrifugation (e.g. CsCl) or column-based binding assays (commercial kit) (Sentchilo et al., 2013; Smalla et al., 2015). Plasmid-safe DNase treatment can also be used to further purify circular plasmids. The purified DNA can then be directly sequenced or first amplified to ensure sufficient plasmid DNA for sequencing. The most common method to amplify plasmids is multiple displacement amplification (MDA), which uses ϕ29 DNA polymerase, an enzyme that amplifies unspecific DNA fragments greater than 70 kb and possesses high fidelity and proofreading activity (Blanco et al., 1989). An alternative is the transposon-aided capture (TRACA) method, which consists of tagging the previously isolated plasmids with a transposon that contains a selectable marker (kanamycin resistance) and the *Escherichia coli* origin of replication (OriV). Subsequent transformation of *E. coli* with these tagged plasmids permits amplification and sequencing (Jones and Marchesi, 2007). Viral particles can also be separated from bacterial cells by CsCl gradient centrifugation, although previous steps such as filtration or precipitation might be necessary to enrich the viral particles in the sample. DNA or RNA is then extracted, amplified if necessary, and sequenced (Thurber et al., 2009).

All of these culture-independent methods have a common disadvantage: the *in situ* bacterial host of the plasmid remains unknown. Several technologies have recently been developed to attempt to solve this problem. (i) Epic-PCR can link functional genes, e.g. a plasmid replicase, with phylogenetic markers in uncultured single cells (Spencer et al., 2016). (ii) Single-cell genomic sequencing (SiC-seq) captures the whole-genome information of a single cell including plasmids and associated viruses (Doud and Woyke, 2017). This method can use droplet microfluidics, optical tweezers or flow sorting to obtain single cells (Zhang and Liu, 2008; Rinke et al., 2014; Lan et al., 2017). (iii) Hi-C technology analyses the 3D folding of genomes. Cells are first fixed with formaldehyde to attach interacting loci by covalent DNA-protein bonds, including interactions of a plasmid with specific parts of the chromosome. The modified DNA is then fragmented by a restriction enzyme and sequenced after other intermediate steps (Van Berkum et al., 2010). As a result, all the chromosomal reads from a specific taxonomic group are binned together, including the plasmids attached to those chromosomal fragments (Burton et al., 2014; Stalder et al., 2019). (vi) Single-molecule real-time (SMRT) sequencing from PacBio Technologies not only determines the sequence of nucleotides but also predicts the methylation of nucleotides. This information is later used to perform a more accurate binning or clustering of contigs belonging to the same taxon. Furthermore, it can link plasmids and other MGEs to their host (Beaulaurier et al., 2018). (v) Identifying MGEs integrated inside bacterial genomes can also help linking them to specific taxa in the binning process during metagenomic analysis. When a MGE enters a bacterial cell, fragments of it are integrated into the genome as spacers of repetitive sequences called Clustered Regularly Interspaced Palindromic Repeats (CRISPR). Thus, it is possible to derive the viruses that have infected a specific host (Shkoporov et al., 2019).

Obtaining high quality and high yields of exDNA is challenging due to its low concentration in wastewater. Furthermore, avoiding cell lysis during exDNA isolation protocols is often unchecked and difficult to achieve. However, throughout the past years, new methods have been developed for exDNA analysis from water and soil samples (Nagler et al., 2018). The most recent techniques applied to wastewater are the use of magnetic beads and chromatography to purify exDNA after filtration of the sample (Yuan et al., 2019; Calderón-Franco et al., 2020). The first method added magnetic beads to 5 mL of sample filtrate to bind the exDNA. After washing the beads to remove impurities, exDNA was eluted for further analysis. This method gave better exDNA yields per sample volume and quality than previous methods such as alcohol precipitation, surfactant cetyl trimethyl ammonium bromide (CTAB) precipitation and DNA extraction kits. However, the authors did not check for cell lysis during the protocol even though sample vortexing was applied. The second method used higher amounts of sample (1000 mL) and passed it through an anion exchange chromatography column. The eluted exDNA was tracked with a UV-VIS absorbance detector and further precipitated and treated with proteinase K. The exDNA yield per volume sample was lower compared to the magnetic beads method, but still good for further molecular and sequencing analysis. Cell lysis was monitored by live-dead staining and flow cytometry analysis. Fortunately, no cell disruption was observed after centrifugation or filtration.

A recent review described in detail many of the above methods for *de novo* MGE identification (Carr et al., 2020). In addition, another study compared some of the above culture-dependent and culture-independent techniques in the analysis of broiler cecal samples (Delaney et al., 2018) to determine which method provided the largest variety of antibiotic resistance plasmids capable of expression in a human pathogenic strain of *Escherichia coli*. The results showed that single commercial kits or alkaline lysis were not effective at obtaining intact plasmids. Using the TRACA method, only a few plasmids were extracted. Although MDA gave the best results, this technique was not as consistent as exogenous plasmid isolation. Thus, the authors recommended using this last technique in order to obtain a comprehensive overview of antibiotic resistance plasmids from a complex sample. This study lacked many of the novel methods described in this review. For that reason, a detailed description of advantages and disadvantages of each method is written in Box 1 and Table 2. If the objective is to study the function of specific MGEs, culture-based methods are needed in order to grow the bacterial cells in the laboratory. However, if the objective is to identify new MGEs or study the full diversity of MGEs in a specific environment, culture-independent methods are needed. The best approach would be the combination of short-read sequencing for accuracy and other technologies such as SMRT or Hi-C for a correct MGE assembly and linkage to host cells. Single-cell sequencing can provide further resolution by distinguishing very closely related bacteria. However, obtaining single cells from biofilms, granules or flocs requires extra optimization of the technique.

## 4 Transfer of catabolic genes as a bioaugmentation strategy in wastewater treatment plants

Traditional bioaugmentation consists of adding OMP-degrading microorganisms to contaminated sites where the indigenous microorganisms do not possess or express the ability to degrade the pollutants (Singer et al., 2005). Although this method has shown success, repeated addition of microorganisms is often needed due to the poor survival and activity of the inoculum (Gilbert and Crowley, 1998). A promising alternative to relying on the survival and activity of the inoculum is to spread degradative genes among the indigenous community. As MGEs are natural vehicles for the rapid dissemination of genes among complex microbial communities, they have been used to accelerate the bioremediation of toxic compounds in various environments such as polluted soils and waters (Top et al., 2002). Catabolic MGEs have been successfully introduced into different WWTP systems in order to enhance the biodegradation of recalcitrant organic compounds (see references in Table 3). However, ensuring the spread and persistence of these degradative genes is usually a great challenge. In the following sections, different (a)biotic factors contributing to MGE transfer and persistence in WWTPs will be examined. Moreover, several MGE-mediated bioaugmentation strategies in WWTPs will be explained, and their limitations and risks will be discussed.

**Table 3:** MGE-mediated bioaugmentation strategies used in WWTP-like settings and aqueous solutions.

Pollutant	Donor	MGE	Transfer	Degradation increase	Set-up	Reference
3-Chlorobenzoate (100 mg/L – 1 g/L)	*Pseudomonas putida* UWC1 (good survival, 8 weeks, 10^4^-10^5^ bacteria /mL)	Plasmid pD10 (non-conjugative IncQ)	Yes	No under activated sludge conditions. Yes in pure cultures.	Activated sludge microcosm	McClure et al. (1989)
Two experiments: 3-chlorobenzoate and 4-methylbenzoate (150–600 mg/L)/4-ethyl benzoate (350 mg/L)	*Pseudomonas* sp. strain B13 FR1/ *Pseudomonas putida* KT2440 (good survival, 2 weeks, 10^4^-10^5^ bacteria /mL)	pFRC20P (non-conjugative but mobilizable plasmid)/pWWO-EB62 (IncP-9 TOL plasmid, self-transmissible)	No/Yes	Yes/No because indigenous bacteria readily degraded 4-ethyl benzoate	Activated sludge microcosm	Nüsslein et al. (1992)
3-Chlorobenzoate (109 mg/L) 1,4-Cichlorobenzoate Toluene	*Pseudomonas* sp. strain B13 (poor survival, it decreased after 2 days to less than 10^5^ bacteria /mL)	Self-transmissible clc element	Yes, to inoculated recipient strain *Pseudomonas putida* F1 and indigenous bacteria	Unknown	Activated sludge microcosm	Ravatn et al. (1998)
3-Chlorobenzoate (467 mg/L sole carbon source)	*P. putida* BN210 (poor survival, disappearance of donor strain)	Self-transmissible clc element	Yes	Yes	Conventional activated sludge system (CAS) and membrane reactor (MBR)	(Ghyoot et al., 2000; Springael et al., 2002)
3-Chloroaniline (150 mg/L)	*Pseudomonas putida* UWC3	Plasmid pC1 (IncP-1 beta, self-transmissible)	Yes	Yes in pure culture but not tested under real activated sludge conditions	Activated sludge microcosm	Goris et al. (2003)
3-Chloroaniline (100 mg/L sole carbon source)	*Pseudomonas putida*	Plasmid pNB2 (IncP-1 beta, self-transmissible)	Yes	Yes	Activated sludge microcosm	Bathe (2004)
2,4-Dichlorophenoxyactic acid (440 mg/L sole carbon source)	*Pseudomonas putida* (poor survival, disappearance of donor strain)	Plasmid pJP4 (IncP-1 beta, self-transmissible)	Yes	Yes	Sequencing batch biofilm reactor inoculated with activated sludge	Bathe et al. (2004)
3-Chloroaniline (80 mg/L)	*Pseudomonas putida* (poor survival, disappearance of donor strain)	Plasmid pNB2 (IncP-1 beta, self-transmissible)	Yes	Yes	Sequencing batch moving bed reactor inoculated with activated sludge	Bathe et al. (2005)
Benzyl alcohol (540 mg/L sole carbon source)	*Pseudomonas putida* KT2442 (good survival, attached to granules)	pWWO (IncP-9 TOL plasmid, self-transmissible)	Yes	Yes	Aerobic granular sludge microcosm	Nancharaiah et al. (2008)
3-Chloroaniline (80–400 mg/L)	*Comamonas testosteroni* (low abundance towards the end of the experiment)	pNB2 (IncP-1 beta, self-transmissible)	Yes	Yes	Semi-continuous activated sludge reactors and biofilm reactors	Bathe et al. (2009)
Benzyl alcohol (54–108 mg/L)	*Pseudomonas putida* (good survival at laboratory scale)	pWWO (IncP-9 TOL plasmid, self-transmissible)	Yes	Yes but only at laboratory scale	Pilot and laboratory scale sequencing batch biofilm reactors	Venkata Mohan et al. (2009)
2,4-Dichlorophenoxyacetic acid (8–385 mg/L sole carbon source and with mixed substrates)	*Pseudomonas putida* (poor survival, disappearance after 30 h)	pJP4 (IncP-1 beta self-transmissible)	Yes	Yes	Aerobic sludge granules in sequence batch reactors and microcosms	(Quan et al., 2010; Ma et al., 2012)
2,4-Dichlorophenoxyactic acid	*Escherichia coli* HB101 or *Pseudomonas putida* KT2440	pJP4 (IncP-1β self-transmissible)	Yes	Unknown	Filter matings with activated sludge microorganisms	Tsutsui et al. (2010)
2,4-Dichlorophenoxyacetic acid (14–500 mg/L sole carbon source and with mix substrates)	*Pseudomonas putida* SM1443 (poor survival, disappearance after 8 days)	pJP4 (IncP-1 beta self-transmissible)	Yes	Yes but biodegradation was reduced when other carbon sources were present	Aerobic sludge granules in sequence batch reactors	Quan et al. (2011)
Dibenzofuran (120 mg/L)	*Rhodococcus* sp. strain p52 (Donor cells decreased 3 orders of magnitude after 80 days)	pDF01 and pDF02 (dioxin catabolic plasmids)	Yes	Yes	Laboratory-scale sequencing batch reactors (SBRs)	Ren et al. (2018)
Naphtalene (2 g/L)	*Pseudomonas putida* ND6	pND6-1 and pND6-2	Yes	Yes	Aquous solutions: phosphate buffer, artificial lake, and oilfield-produced water	Wang et al. (2020b)
Phenol	*Pseudomonas putida*	pEST1024	Yes (creation of new phenol-degrading plasmids)	Unknown	Phenol-polluted rivers	Elken et al. (2020)
Pesticides	*Pseudomonas putida* KT2440	RP4, RSF1010, pKJK5 and pWWO (IncP-9 TOL plasmid, self-transmissible)	Yes	Unknown	Filter matings with microorganisms from groundwater sand filters	Pinilla-Redondo et al. (2020)

### 4.1 Conditions affecting conjugative gene bioaugmentation in wastewater treatment plants

Selecting a successful gene augmentation strategy in complex microbial ecosystems such as WWTPs depends on finding a proper match among the bacterial donor strain, competent environmental recipients, the MGE carrying the gene(s) of interest, and the (a)biotic conditions in the WWTP reactors. Only conjugative gene transfer has been reported to be effective thus far. Conjugation occurs more efficiently when high numbers of donor and recipient strains are in close contact through mating aggregates (Ravatn et al., 1998). These aggregates are structures that contain physically bound cells exchanging DNA material. In WWTPs, mating aggregates can be granules or colonizable surfaces where microorganisms can form biofilms. The suitability of flocs for mating is questionable due to their porous nature. As the biomass density in water increases, so does the probability of conjugation events. No study has attempted to introduce genes into the environment using viral vectors as in human gene therapy (Bramson et al., 1995). One reason is the presumed high specificity of bacteriophages for bacterial infection, which might reduce the usefulness of transduction for gene exchange among distantly related bacteria (Dröge et al., 1999). However, recent studies suggest that phages with broad host ranges are very common in nature (de Jonge et al., 2019), supporting the potential of bacteriophages for developing new gene augmentation strategies in WWTPs.

One factor determining the effectiveness of conjugative gene transfer is the initial stability and growth of the **donor strain**. The establishment of the donor strain in the WWTP microbial community is not a requirement for transfer of the catabolic MGE, but high initial numbers of donor bacteria increase the chances of gene transfer (Ravatn et al., 1998). The best approach is to select indigenous bacteria as donor cells because they are already adapted to the physical, chemical, and biological conditions of the polluted environment (McClure et al., 1991). However, this might be a challenge, as environmental bacteria are often difficult to genetically modify or grow in the lab. Moreover, for obvious reasons, donor strains should not be pathogenic. The suitability of the donor bacteria also depends on the a(biotic) conditions in the WWTPs: donor cells must be able to survive at the temperature, oxygen concentration, pH, and other parameters present in the activated sludge basins (Merlin et al., 2011). Furthermore, they must successfully compete with indigenous microorganisms and avoid predation by protozoa and bacteriophages (Dröge et al., 1999; Mrozik and Piotrowska-Seget, 2010). Fulfilling these criteria will guarantee the initial stable cell number necessary to achieve effective gene transfer.

Once the donor cells are stably introduced, the **transfer of catabolic genes** to other recipient bacteria can be initiated. Many biotic and abiotic variables have an effect on conjugation efficiency and should be considered when designing MGE-mediated bioaugmentation strategies (Dröge et al., 1999). One important factor is the type of **MGE** chosen for catabolic gene dissemination. In aqueous environments, DNA transfer through plasmids encoding flexible pili is more effective. Furthermore, broad-spectrum plasmids are better vectors for spreading the associated genes among the microbial community, even to distantly related bacteria. Environmental or abiotic factors such as temperature and the presence of toxic compounds also affect conjugation (Dröge et al., 1999). In fact, subinhibitory concentrations of antibiotics, heavy metals, disinfectants or other organic compounds can stimulate the transfer of ARGs via several MGEs including transformation (Merlin et al., 2011; Jiao et al., 2017; Zhang et al., 2017, 2018; Wang et al., 2020c).

When genes for the degradation of toxic compounds are horizontally transferred, the metabolic reactions they encode must function in biochemical and regulatory environments that they did not coevolve with. Consequently, **post-transfer genetic refinement** of the recipient cell may be necessary. In a previous study, researchers introduced the gene for dichloromethane (DCM) degradation in *Methylobacterium* strains that had not been previously exposed to the contaminant. They observed that the new recombinant strains had difficulty growing on DCM, and they hypothesized that the toxic by-products generated from DCM breakdown were responsible (Michener et al., 2014a, Michener et al., 2014b). In a subsequent experiment, they proved that adaptation to growth on DCM involved mutations that increased chloride excretion from the cell. Furthermore, they constructed a new MGE containing both a DCM degradation pathway and a chloride exporter and achieved successful DCM degradation in recombinant bacteria (Michener et al., 2014a, Michener et al., 2014b). These results demonstrate the importance of understanding microbial adaptation to OMP degradation in order to design a proper catabolic MGE (de Lorenzo et al., 2015).

Once the desired genes are successfully spread among indigenous bacteria and are functional, the ultimate goal is to make these genes persist in WWTPs and thus achieve a constant OMP degradation rate. Selective pressure such as OMP presence is the key factor controlling the persistence of plasmid-bearing bacteria. As long as the plasmid-bearing bacteria have a fitness advantage compared to plasmid-free bacteria, the catabolic MGEs will persist. One example of a clear advantage for donors and plasmid-bearing bacteria is when all carbon and nitrogen have been removed and OMPs prevail in WWTP settling tanks. A previous study demonstrated that the selection and growth of donor and transconjugant cells were dependent on the presence of sufficiently high concentrations of the specific substrate (Ravatn et al., 1998). To predict the long-term dynamics of catabolic plasmids in a polluted environment, a mathematical model was developed that considered the negative feedback between plasmid spread and selective advantage: when catabolic genes are highly spread, the OMP concentration will decrease faster, thereby lowering the selective advantage of plasmid-bearing bacteria (Miki et al., 2007). Thus, in order to achieve long-term OMP degradation, it is necessary to find the right plasmid spread efficiency in order to maintain constant, low pollutant concentrations, as the degrading microbial community remains more stable when OMP degradation occurs slowly.

### 4.2 Cases of gene bioaugmentation under wastewater treatment plants conditions

The increasing sensitivity of mass spectrometry detection methods is constantly revealing new emerging contaminants in WWTPs. Although catabolic MGEs are known to play a relevant role in adaptation to such new toxic compounds, few studies have successfully used MGE-mediated bioaugmentation strategies to increase their biodegradation in WWTPs (Table 3). In this section, past attempts using conjugation to spread degradative genes under WWTP settings are evaluated, and their limitations and risks are discussed.

The pollutants used in these experiments (Table 3) were industrial compounds such as chlorobenzoic acid, the herbicide 2,4-dichlorophenoxyacetic acid (2,4-D), and pesticides. All were investigated at concentrations of mg/L, although they often appear in the range of ng/L to μg/L, except for industrial WWTPs where concentrations can be higher. This suggests an important question: do these low concentrations elicit sufficient selective pressure to achieve successful gene augmentation solutions in urban WWTPs? Furthermore, many experiments were performed using the pollutant as the sole carbon source. One study observed that biodegradation of 2,4-D was higher when it was the only carbon source than when other more easily degradable carbon sources were present, probably because of catabolite repression (Quan et al., 2011). These experimental designs outline the need for more realistic pollutant concentrations and influent compositions in test bioreactors.

The most commonly used donor strain was *Pseudomonas putida* (Table 3), probably due to the availability of molecular tools for genetic engineering of this microorganism and its presence as an environmental bacterium in WWTPs and soils around the globe (Nikel et al., 2016). In more recent studies, *Comamonas testosteroni*, *Escherichia coli*, and *Rhodococcus* sp. were also applied (Bathe et al., 2009; Tsutsui et al., 2010; Ren et al., 2018). Different MGEs were used, mainly plasmids. Most were self-transmissible and had broad host ranges, thus resulting in successful transfer of catabolic genes among the studied community, usually in activated sludge. In some cases, the donor strain disappeared within a few days after application. However, this did not hamper the success of the bioaugmentation because the catabolic MGE had successfully been transferred to indigenous bacteria persisting in the environment. In many of the studies in Table 3, fluorescent proteins were used to label the donor strain and the MGE with different colors (green fluorescent protein and DsRed). The use of these genetic markers with confocal laser scanning microscopy provides an effective and rapid way to visually determine if gene transfer to indigenous bacteria has occurred and if donor bacteria are still present (Nancharaiah et al., 2008; Venkata Mohan et al., 2009; Quan et al., 2010, 2011).

Many of the studies in Table 3 were successful in improving pollutant degradation by gene augmentation according to three criteria: (i) increased degradation of the compound compared to non-bioaugmented microcosms or bioreactors, (ii) transfer of the catabolic MGE to indigenous bacteria, and (iii) decreased donor strain abundance after some time. This success clearly supports the potential of gene augmentation to overcome the donor strain survival problem of traditional bioaugmentation. All of the experiments in Table 3 with promising results were performed in lab-scale reactors or microcosms mimicking activated sludge conditions. The only gene augmentation trial in a pilot reactor failed (Venkata Mohan et al., 2009), which indicates that unknown challenges in the scale up of gene bioaugmentation strategies remain and suggests the need for more experiments in on-site reactors at WWTPs (Nzila et al., 2016).

In order to choose the right donor and MGE, more knowledge is needed about which catabolic MGEs are being transferred in WWTPs and which bacteria are involved in the process. Furthermore, whether concentrations in the range of ng/L and ug/L are sufficient to create a selection pressure for OMP-degrading bacteria needs to be tested. New contaminants such as diclofenac have been included in watch lists all around the world (Carvalho et al., 2015), so bioaugmentation experiments with these compounds are also needed. In general, more settings that realistically mimic WWTPs are required to optimize conjugation as a realistic solution. Furthermore, new technologies such as viral transfer also hold promise.

The application and generation of genetically-engineered microorganisms (GEMs) in open contaminated sites such as soils or WWTPs remains controversial. The societal concerns towards gene bioaugmentation strategies are related to the unknown consequences of microorganisms with new functions in an open environment (Trump et al., 2020). First, these microorganisms can be spread to other environments with unforeseeable effects. Second, the enhanced transfer of catabolic genes can also increase the mobilization of ARGs. A careful assessment of the risk/benefit ratio is essential to coherent decision-making. On the one hand, a clear benefit decreasing OMP discharge and thus decreasing ecotoxicity in superficial and ground waters can be obtained. On the other hand, new studies assessing gene bioaugmentation risks such as the transfer of ARGs have to be performed. Since self-transferable MGEs are preferred in gene augmentation strategies, they might act as mobilizing agents of other MGEs containing ARGs. Past bioremediation strategies in contaminated waters or soils tried to control GEMs by inserting suicidal genes that kill them after the contaminant disappeared (Molin, 1993). However, this is contrary to bioaugmentation in WWTPs since the contaminant concentrations fluctuates and the objective is to keep the transformed community stable. Other genetic safeguards restrict the GEMs to defined environments or prevent HGT to environmental bacteria (Asin-Garcia et al., 2020). Since gene-augmentation strategies rely on HGT, the only option is to contain GEMs in the WWTPs bioreactors. However, it seems quite difficult to find a way of containing all new transformants inside the WWTP. In conclusion, risk assessment studies are necessary in order to identify associated spreading of genes such as ARGs and to detect GEMs esscaping from WWTPs. In this way, new safeguards strategies can be added to diminish the specific risks.

## 5 Concluding remarks

Microorganisms have been in contact with OMPs for a relatively short period on an evolutionary time scale, so the metabolic pathways are still being developed. During the adaptation process, MGEs provide reliable scaffolds for clustering the genes responsible for OMP degradation and spreading them among bacterial communities. Consequently, OMP degradation genes are often linked to MGEs. In this review, we assessed the prevalence, detection methods, and bioremediation uses of catabolic MGEs in WWTPs:

1) The majority of catabolic MGEs found in WWTPs are conjugative IncP-1 plasmids and IS*1071* insertion sequences affiliated with *Burkholderiales* bacteria. However, this information is biased because many of these studies used culture-based methods. To obtain a realistic overview of the catabolic MGEs with the greatest ecological relevance in WWTPs, it is necessary to move towards cultivation-independent methods with the potential for sequencing all MGEs and associated genes present in environmental samples.
2) There are three main challenges for a thorough study of mobile degradative genes via cultivation-independent methods: (i) the complexity of MGEs assembly from a metagenomic sample because of the long repetitive sequences, (ii) the lack of knowledge of genes encoding enzymes for OMP biodegradation, and (iii) the difficulty in identifying the host microorganisms. So far, the combination of short-read sequencing (for high sequence accuracy) and SMRT or Hi-C (for better assembly and host cell prediction) provides the best option to get a comprehensive representation of MGEs in environmental samples. In addition, more functional analyses are needed to discover new biodegradation genes.
3) MGEs can be used to spread degradative capabilities in WWTPs, thus overcoming the main limitation of traditional cell bioaugmentation: the low survival of the inoculum. Several studies have successfully implemented this technique in lab-scale reactors and activated sludge microcosms. However, gene augmentation failed when scaling up to a pilot reactor. Furthermore, the OMP concentration in these experiments was mg/L, whereas concentrations of ng/L and ug/L are typically found in the influent of urban WWTPs. For these reasons, further research is needed using more realistic scenarios, including pilot WWTP reactors and lower concentrations of OMPs. Risk assessment studies looking at the fate of transformants and the spread of ARGs are also necessary. Hopefully, solving these issues will lead to a safe, cost-effective, and eco-friendly technology that enhances OMP removal in WWTPs.

## Contributors

ABRM conducted the literature search and critical analysis. All authors contributed to structuring and writing the article.

## Declaration of competing interest

The authors declare that they have no known competing financial interests or personal relationships that could have appeared to influence the work reported in this paper.
